# MicroRNA-708 targeting ZNF549 regulates colon adenocarcinoma development through PI3K/AKt pathway

**DOI:** 10.1038/s41598-020-73929-w

**Published:** 2020-10-07

**Authors:** Zhidong Zhao, Xianju Qin

**Affiliations:** Shanghai Eighth People’s Hospital, Shanghai, 200235 China

**Keywords:** Colon cancer, Tumour biomarkers

## Abstract

Colon adenocarcinoma (COAD) is the most common type of gastrointestinal cancer and is still the third leading cause of cancer-related mortality worldwide. Therefore, finding new and promising drugs to eradicate cancer may be a feasible method to treat COAD patients. Cys2-His2 zinc finger proteins (ZFPs) is one of the largest transcription factor family and many of them are highly involved in regulation of cell differentiation, proliferation, apoptosis, and neoplastic transformation. In this study, we identified a tumor-inhibiting factor, ZNF549, which expressed lowly in COAD tissues and COAD cell lines (HT29, HCT116, SW480, LoVo, and SW620). Overexpression of ZNF549 inhibit the ability of COAD cell proliferation and migration. On the contrary, decreasing the ZNF549 expression level promote the ability of COAD cell proliferation and migration. Through bioinformatics analysis, we found that ZNF549 was a potential target of hsa-miR-708-5p (miR-708-5p). Furthermore, we verified the possibility of miR-708-5p targeting the ZNF549 gene, and miR-708-5p inhibited the expression of ZNF549 by luciferase reporter assays, qRT-PCR and western blot assays. Moreover, the relationship between miR-708-5p and phosphatidylinositol 3-kinase/AKt (PI3K/AKt) signal pathway was elucidated. Overexpression and inhibition of miR-708-5p resulted in increased and decreased expression of p-AKt and p-PI3K in HCT116 cells, respectively. RT-qPCR and western blot assays results demonstrated that miR-708-5p regulated COAD cells development by promoting the process of Epithelial-mesenchymal transition (EMT) through PI3K/AKt signaling pathway. In summary, our findings demonstrated that ZNF549, the target gene of miR-708-5p, functions as a tumor suppressor to inhibit COAD cell lines proliferation and migration through regulate the PI3K/AKt signal pathway.

## Introduction

In terms of occurrence, colon cancer is currently the most common type of gastrointestinal cancer and remains the leading cause of cancer-related mortality worldwide^1,2^. The most common subtype of colon cancer is colon adenocarcinoma (COAD) which accounts for 98% of newly diagnosed colon cancer cases^3^. The development of COAD is a multi-stage process, identifiable at the level of histopathology. Normal mucosa turns into adenoma, and then turns into carcinoma^4,5^. The most common reason for COAD treatment failure is recurrence and metastasis^5^. The overall 5-year survival rate for COAD, particularly for patients at advanced stages, is less than 38%^6^. Through a large number of translation regulation studies, the relationship between various molecular markers in COAD and promising clinical results has been evaluated, but the molecular mechanism of this malignant tumor were not comprehensive.

The Cys2/His2-type zinc-finger proteins, belonging Zinc-finger (ZF) proteins family, are widely distributed in humans, and some of them have been found to be involved in cancer occurrence and development^7,8^. In vitro and in vivo studies have shown that miR-940 promotes gastric carcinoma metastasis by down-regulating a direct target gene, a potential metastasis suppressor gene ZNF24^9^. The results provide a clear understanding of the underlying mechanism of gastric carcinoma. In addition, compared with normal tissues, ZNF367 expression level is higher in malignant paraganglioma, adrenocortical carcinoma and thyroid cancer^10^. The ZNF259 expression was lower in the lung cancer tissues compared to adjacent normal lung tissues by immunohistochemical staining and western blot assays^11^. What’s more, ZNF259 inhibits non-small cell lung cancer cells proliferation and invasion by FAK-AKT signaling pathway^11^. However, ZNF549 involved in cancer occurrence and development have not been reported, especially COAD.

MicroRNAs (miRNAs) are a class of highly conserved, single-stranded, small (20 nt–24 nt) noncoding RNA molecules involved in the gene expression regulation^12^. Since each miRNA can regulate the expression of hundreds of target mRNAs to inhibit gene expression or induce mRNA degradation, miRNAs can multifunctional as master coordinators, efficiently regulating fundamental cellular processes, including proliferation, development, and apoptosis^13,14^. Cumulative findings have revealed the miRNAs also play an important role in human carcinogenesis, tumor progression, and metastasis. For instance, miR-296 relative expression level decreased in cervical cancer tissues and cell lines, and can directly targets specificity protein 1 (SP1) to suppress cell proliferation and invasion^15^. MiR-449a regulates cell proliferation, migration and invasion by targeting high mobility group box 1 (HMGB1). It has been found that HMGB1 is overexpressed and is involved in the pathogenesis of various human cancers^16^. Furthermore, miR-847 and miR-329 are involved in metastasis of gastric carcinoma by inhibiting STAT3/vascular endothelial growth factor (VEGF)-A and T lymphoma invasion and metastasis 1 (TIAM1), respectively^17,18^.

Previous studies have demonstrated that miR-708 act as tumor suppressor or oncogene during multiple processes in cancer. Compared with the control group, the expression of miR-708 significantly reduced in glioblastoma cell lines A172, T98G, U87, and U251. This study suggests that miR-708 may play an important tumor suppressor role in glioblastoma^19^. In renal cancer cells, miR-708 induced apoptosis and suppressed tumorigenicity^20^. On the contrary, increased miR-708 expression is associated with poor prognosis in lung adenocarcinoma^21^. Thus it can be seen, the function of miR-708 is associated with the cancer type. Previously, we found that miR-708 overexpressed in COAD patients with early recurrence in the online cancer OMICS database UALCAN in our research. However, the biological roles and target genes of miR-708 in COAD metastasis remains unclear.

In this study, we analyzed the function and molecular mechanism of ZNF549 in COAD tissues and cells. The results demonstrated that ectopic expression of ZNF549 in the COAD cell lines resulted in increased proliferation and migration. What’s more, we also confirmed that ZNF549 was a direct target gene of miR-708-5p in COAD with dual-luciferase reporter assays. RT-qPCR and western blot assays results demonstrated that miR-708-5p regulated COAD cells development by promoting the process of EMT through PI3K/AKT signaling pathway.

Based on our results, we conclude that miR-708-5p promotes the proliferation of COAD cells by mediating the regulation of PI3K/Akt pathway. Based on our results, we deduced that miR-708-5p promoted COAD cells proliferation by mediating the regulation of PI3K/Akt pathway. Exploring the anticancer role of ZNF549 in COAD may contribute to the development of novel therapeutic strategies for COAD cancer patients.

## Results

### ZNF549 is downregulated in COAD tissues and cell lines

**Figure 1:**
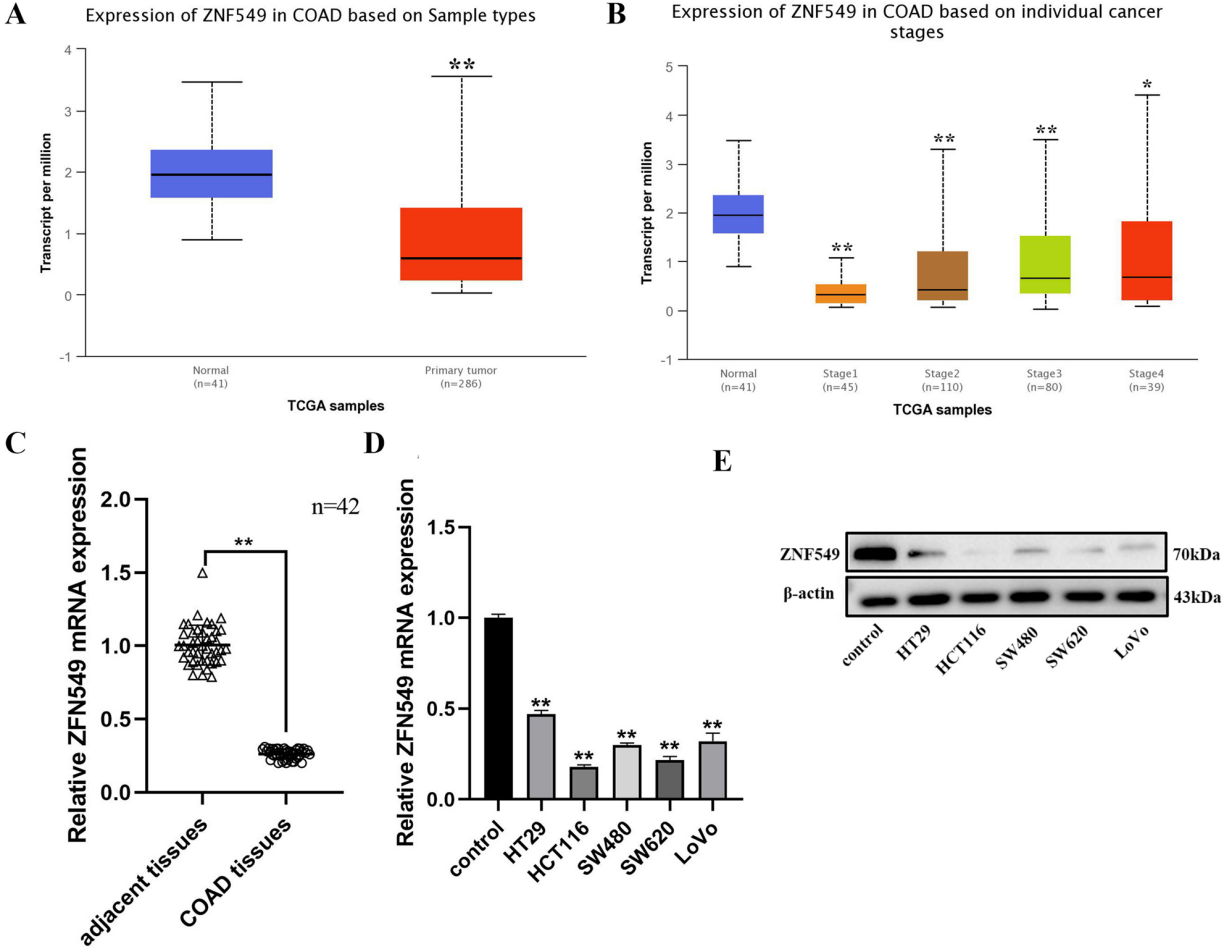
Low expression level of ZNF549 in COAD tissues and cell lines. (**A**) Relative expression of ZNF549 in normal (n = 41) and colon adenocarcinoma (COAD) samples (n = 286) based on sample types from online cancer OMICS database UALCAN (https://ualcan.path.uab.edu/cgi-bin/TCGAExResultNew2.pl?genenam=ZNF549&ctype=COAD). **p < 0.01. (**B**) Relative expression of ZNF549 in normal and COAD samples based on individual cancer stages from online cancer OMICS database UALCAN (https://ualcan.path.uab.edu/cgibin/TCGAExResultNew2.pl?genenam=ZNF549&ctype=COAD).  **p < 0.01, *p < 0.05. (**C**) Relative expression of ZNF549 in 42 paired COAD tissues and COAD adjacent tissues. *β-actin* was used as a control. The results were obtained from three independent experiments. **p < 0.01. GraphPad Prism 8.0 software was used for statistical analysis and generating the image. (**D**) The expression level of ZNF549 in COAD cell lines (HT29, HCT116, SW480, SW620, and LoVo). Control was MD2 cell line. **p < 0.01. Data are given as means ± SD (n = 3). The relative expression level were analyzed via the 2^−ΔΔCt^ method. Asterisks indicate significant differences of the amounts between COAD cell lines and the control by Student’s *t*-test. **p < 0.05. *β-actin* was used as internal control. Supplementary Fig. S1 is the full-length originals figure. Student’s *t*-test were used to analyze the significance between groups by GraphPad Prism 8.0 software.

To investigate the potential role of ZNF549 in colon adenocarcinoma (COAD), the ZNF549 expression pattern in COAD samples was evaluated using database from online cancer OMICS database UALCAN (https://ualcan.path.uab.edu/index.html). The results indicated that a significantly lower expression level of ZNF549 was detected in COAD samples than that in corresponding normal tissues (**p < 0.01) (Fig. 1A and Supplementary Table S1A,B). The expression of ZNF549 at different COAD development stages showed in Fig. 1B. It is interesting to note that the expression of ZNF549 increased persistently from stage1 to stage4 (*p < 0.05 **p < 0.01) (Fig. 1B). RT-qPCR analysis were conducted to detect ZNF549 expression level in 42 paired COAD cancer tissues and matched adjacent tissues, as well as COAD cancer cells (HT29, HCT116, SW480, SW620, and LoVo) and MD2 cell (control). The results demonstrated that ZNF549 was downregulated in COAD tissues and cells compared with adjacent tissues and MD2 cell, respectively (**p < 0.01) (Fig. 1C,D). Western blot experimental results showed that the expression level of ZNF549 in COAD cells is also significantly lower than that of control cells (Fig. 1E). Since HCT116 and SW620 were more significantly reduced compared to other cell lines, we used these two cell lines for further analysis. In conclusion, these results suggested that ZNF549 may play important role in the formation and progression of COAD.

### ZNF549 negative regulate COAD cell proliferation and invasion in COAD cells

**Figure 2:**
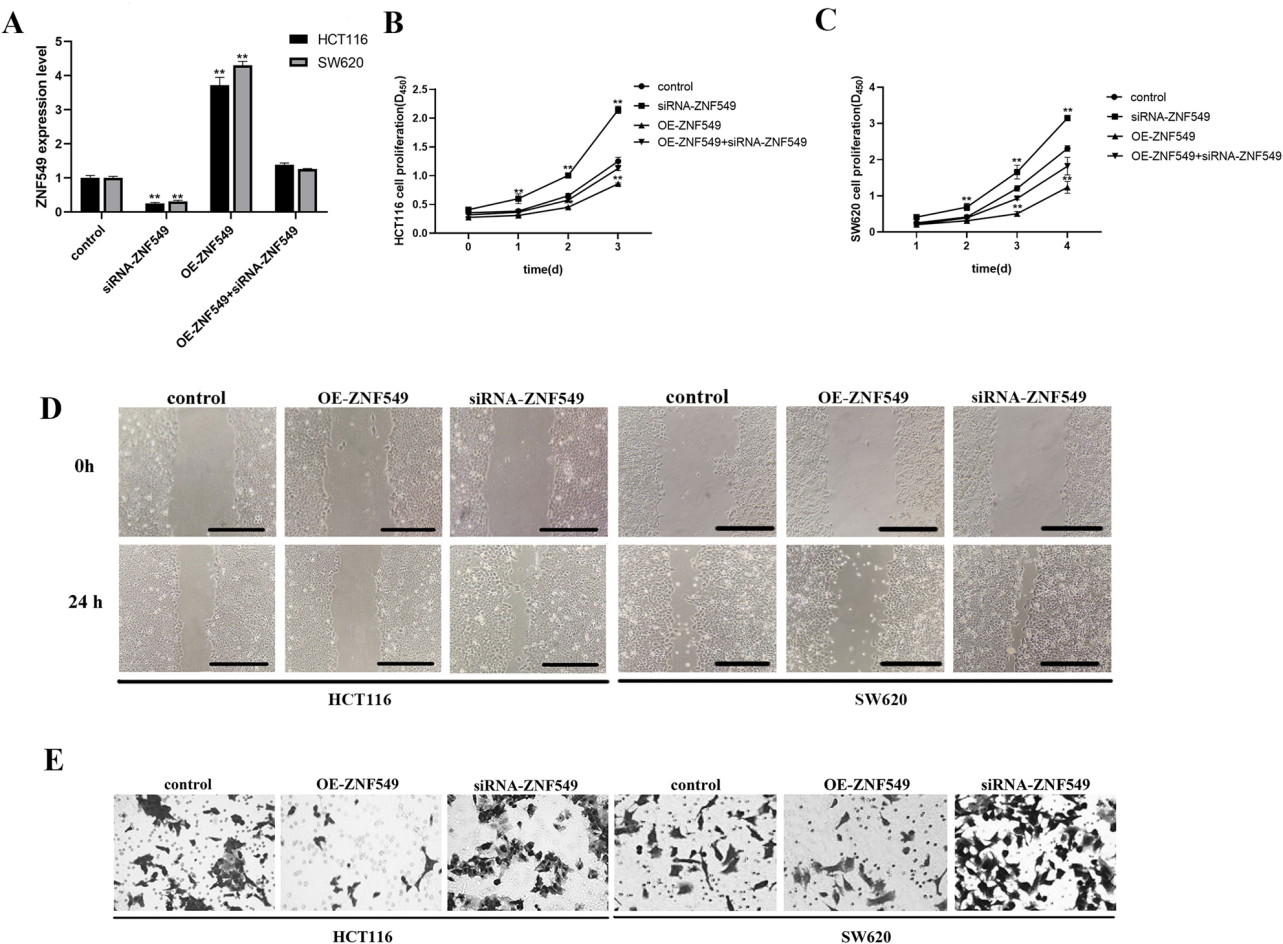
ZNF549 negative regulate cell proliferation and invasion in COAD HCT116 and SW620 cells. (**A**) Relative expression of ZNF549 were assessed in HCT116 and SW620 cells after transfection with OE-ZNF549, siRNA-ZNF549 and OE-ZNF549 + siRNA-ZNF549 by qRT-PCR. **P < 0.05. (**B**, **C**) Cell proliferation viability in HCT116 and SW620 cells after transfection with OE-ZNF549, siRNA-ZNF549 and OE-ZNF549 + siRNA-ZNF549. **P < 0.05; (**D**) Cell migration ability analyses in HCT116 and SW620 cells after transfection with OE-ZNF549, siRNA-ZNF549 and OE-ZNF549 + siRNA-ZNF549. (**E**) Cell invasion analyses in HCT116 and SW620 cells after transfection with OE-ZNF549, siRNA-ZNF549 and OE-ZNF549 + siRNA-ZNF549. Data are given as means ± SD (n = 3). Asterisks indicate significant differences of the amounts between COAD HCT116 and SW620 cell lines and the control by Student’s *t*-test. **P < 0.05. *β-actin* was used as internal control. Student’s *t*-test were used to analyze the significance between groups by GraphPad Prism 8.0 software.

To explore the roles of ZNF549 in COAD. OE-ZNF549, siRNA-ZNF549 and OE-ZNF549 + siRNA-ZNF549 were transfected into HCT116 and SW620 cell lines. RT-qPCR results demonstrated that ZNF549 expression level significantly increased in OE-ZNF549 cells. Conversely, ZNF549 expression level significantly decreased in siRNA-ZNF549 cells (**p < 0.05) (Fig. 2A). CCK-8 results showed that OE-ZNF549 cells proliferation ability was significantly lower than that of the control group (**p < 0.05) (Fig. 2B,C). The proliferation ability of siRNA-ZNF549 cells was significantly higher than that of the control group (**p < 0.05) (Fig. 2B,C). Cell scratch and invasion assays results showed that the OE-ZNF549 cells migration ability was significantly lower than that of the control group. The siRNA-ZNF549 cells migration capacity was opposite (Fig. 2D,E). These results suggested that ZNF549 negative regulate the COAD cells development.

### ZNF549 is a direct target of miR-708-5p in COAD HCT116 and SW620 cells

**Figure 3:**
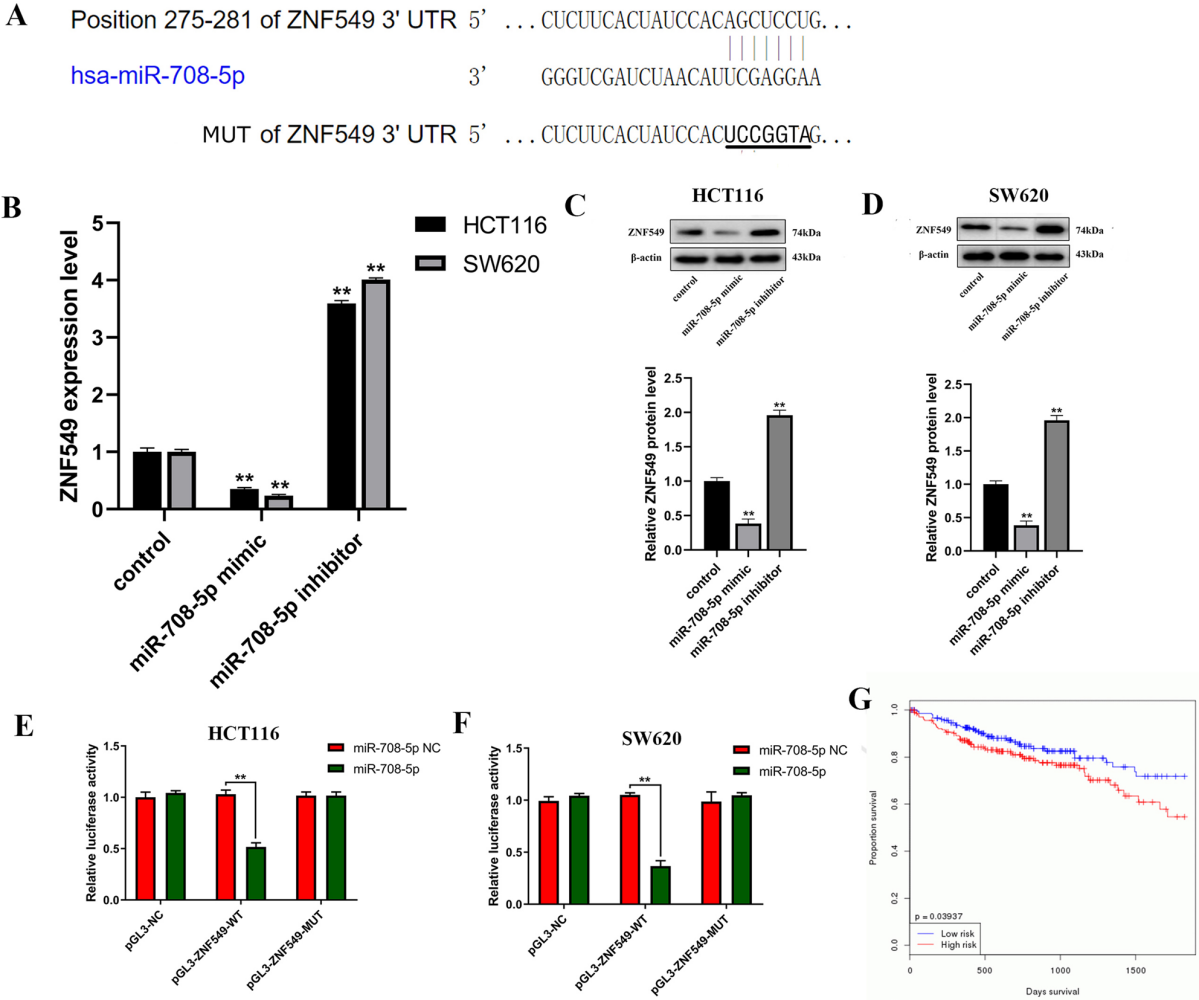
ZNF549 is a direct target of miR-708-5p in COAD HCT116 cells. (**A**) The 3′UTR of ZNF549 mRNA contains the binding sequences of miR-708-5p according to website (https://www.targetscan.org/cgi-bin/targetscan/vert_72/view_gene.cgi?rs=ENST00000594943.1&taxid=9606&members=miR-21-5p/590-5p&showcnc=0&shownc=0&showncf1=&showncf2=&subset=1); (**B**) Relative expression level of ZNF549 in HCT116 and SW620 cells transfected with control/miR-708-5p mimics/miR-708-5p inhibitor. (**C**) ZNF549 protein expression in HCT116 cells transfected with control/miR-708-5p mimics/miR-708-5p inhibitor. (**D**) ZNF549 protein expression in SW620 cells transfected with control/miR-708-5p mimics/miR-708-5p inhibitor. (**E**) Luciferase activities were measured using a dual-luciferase assay system in HCT116 cells. (**F**) Luciferase activities were measured using a dual-luciferase assay system in HCT116 cells. (**G**) Kaplan–Meier curves of OS (overall survival, OS) for COAD patients with miR-708-5p according to website (https://www.oncomir.org/oncomir/survival_custom.html). Data are given as means ± SD (n = 3). Asterisks indicate significant differences of the amounts between COAD HCT116 and SW620 cell lines and the control by Student’s *t*-test. **P < 0.05. *β-actin* was used as internal control. Supplementary Figure S1 is the full-length originals figure. Student’s *t*-test were used to analyze the significance between groups by GraphPad Prism 8.0 software.

To investigate the molecular mechanism on how ZNF549 negative regulate the COAD HCT116 and SW620 cells proliferation and invasion, we carried out the bioinformatics analysis using the TargetScan website (www.targetscan.org). The results indicated that ZNF549 gene transcript 3′-UTR region might be a potential target gene for miR-708-5p (Fig. 3A). As shown in Fig. 3B, miR-708-5p can negative regulate ZNF549 expression level. Additionally, the total protein abundance of ZNF549 increased by miR-708-5p overexpression and decreased by knockdown of miR-708-5p in HTC116 and SW620 cells (Fig. 3B,C). To test whether ZNF549 was a direct binding for miR-708-5p, 3′-UTR fragments of ZNF549 and corresponding mutant counterpart were directly fused downstream of the firefly luciferase gene (Fig. 3A). The results revealed that luciferase activities significantly decreased by miR-708-5p in pGL3-ZNF549-WT transfected COAD HTC116 and SW620 cells (Fig. 3E,F). Kaplan–Meier curves demonstrated that miR-708-5p could promote tumor development (Fig. 3G). In conclusion, miR-708-5p can inhibit the expression of ZNF549 and promote COAD cells development.

### MiR-708-5p promotes EMT through regulating PI3K/AKt pathway in HCT116 cells

**Figure 4:**
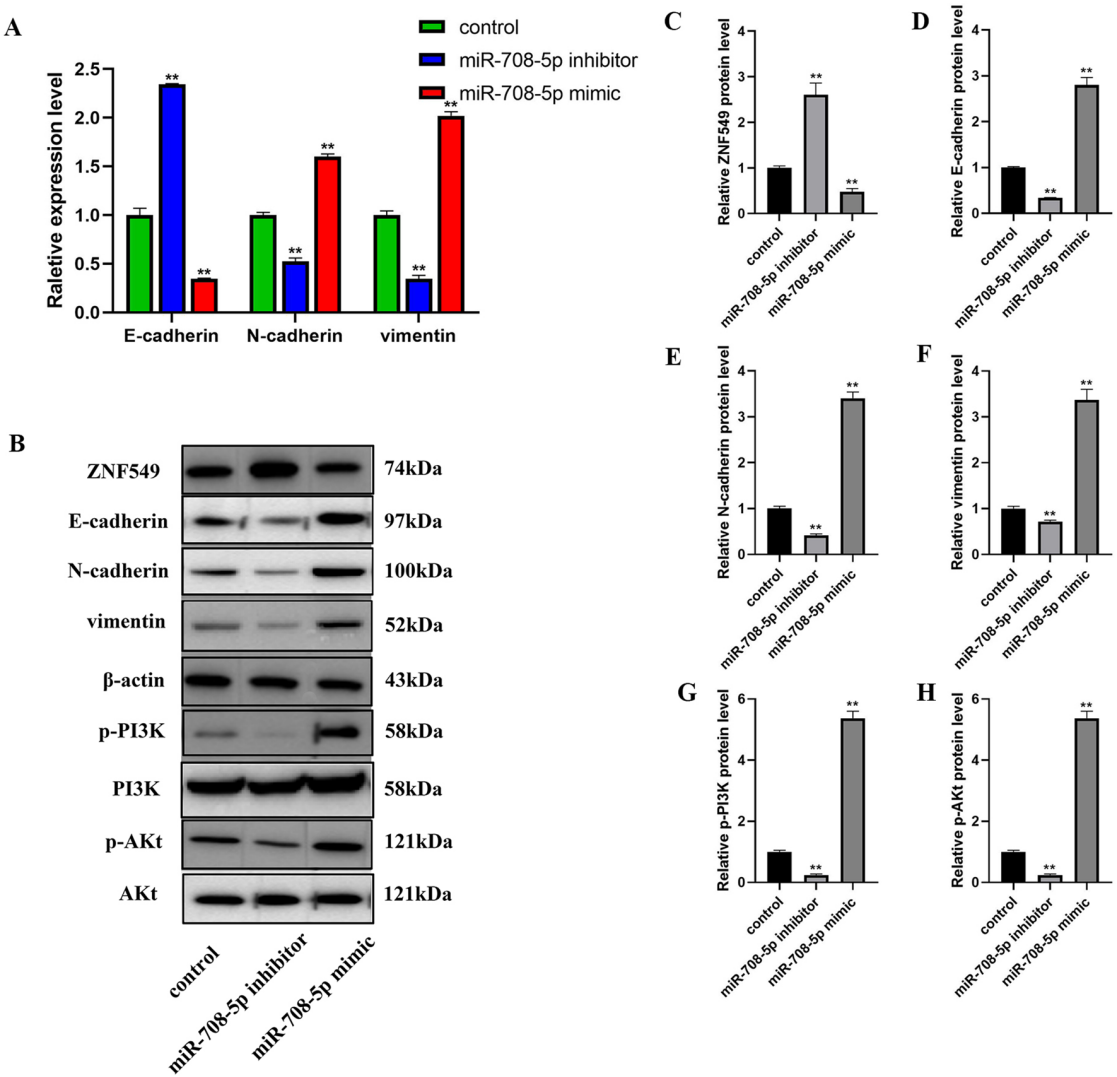
MiR-708-5p regulates development of COAD by promoting the process of EMT through PI3K/AKt signaling pathway. (**A**) Relative expression of EMT related genes in HCT116 cells transfected with control/miR-708-5p mimics/miR-708-5p inhibitor; (**B**) EMT and PI3K/AKT pathway related proteins expression in HCT116 cells transfected with control/miR-708-5p mimics/miR-708-5p inhibitor by western blot assays. (**C**–**H**) ZNF549, EMT and PI3K/AKT pathway related proteins expression level in HCT116 cells transfected with control/miR-708-5p mimics/miR-708-5p inhibitor. Data are given as means ± SD (n = 3). Asterisks indicate significant differences of the amounts between COAD HCT116 cell lines and the control by Student’s *t*-test. **P < 0.05. *β-actin* was used as internal control. Supplementary Fig. S1 is the full-length originals figure. Student’s *t*-test were used to analyze the significance between groups by GraphPad Prism 8.0 software.

Mir-708-5p negative regulate ZNF549 to promoted COAD HCT116 and SW620 cells proliferation and migration, which led us to examine if miR-708-5p had any influence involved in EMT process in HCT116 cells. Using RT-qPCR assays, our results demonstrated that overexpression of mir-708-5p reduced epithelial cell marker gene (E-cadherin) expression, on the contrary, increased mesenchymal cell marker genes (N-cadherin, Vimentin) expression (Fig. 4A), whereas knockdown of mir-708-5p showed opposite effects HCT116 cells (Fig. 4A). At the same time, corresponding results obtained by western blot analysis (Fig. 4B). MiR-708-5p positively regulated the ZNF549 protein level (Fig. 4B,C), whereas negatively regulated E-cadherin (Fig. 4B,D), N-cadherin (Fig. 4B,E), vimentin (Fig. 4B,F), p-PI3K (Fig. 4B,G), and p-AKt (Fig. 4B,H) protein levels. In conclusion, the results suggested that miR-708-5p promoted EMT process through regulating PI3K/AKt pathway in HCT116 cells.

## Discussion

Many researchers are studying the function of ZNF proteins in human cancer^22^. ZNF143 is one of the zinc finger proteins, which enhanced gastric cancer cell migration by promoting the process of EMT through PI3K/AKt signaling pathway^23^. It has been found that ZNF143 participates in the transcriptional regulation of many protein coding genes^24^. Meenu et al., determined the mechanism of gene expression regulation and function of ZNF367 in a variety of endocrine cancers, including adrenocortical carcinoma, papillary thyroid cancer, and pheochromocytoma^10^. Western blotting and qRT-PCR assays showed that ZNF268 was highly expressed in human ovarian cancer, and cell migration experiments showed that ZNF268 gene increased skov-3 cell growth by promoting cell cycle^25^. However, the regulating function and mechanisms of ZNF549 in cancer disease, especially colon adenocarcinoma (COAD) remains unclear and highly controversial.

UALCAN is a comprehensive, user-friendly, and interactive web resource for analyzing cancer OMICS data. It is built on PERL-CGI with high quality graphics using javascript and CSS (Cascading Style Sheets, CSS). UALCAN has been now visited over 300,000 times by cancer researchers from over 100 countries and cited over 500 times. In the present study, we identified a transcription factor, ZNF549 by online cancer OMICS database UALCAN (https://ualcan.path.uab.edu/index.html), which contains COAD clinical information, genomic characterization data, and high level sequence analysis. ZNF549 (zinc finger protein 549), belonging to the kruppel C_2_H_2_-type zinc-finger protein family, is a 640 amino acid nuclear protein that exists as 2 alternatively spliced isoforms and may be involved in transcriptional regulation in cancer disease^26^. The results indicated that a significantly lower expression level of ZNF549 detected in COAD samples than that in corresponding normal tissues (Fig. 1A). Overexpression and repression of ZNF549 resulted in a decrease and increase of proliferation and migration ability in HCT116 cells, respectively (Fig. 2B–D). This is the first time to report that ZNF549 can inhibit the COAD cells proliferation and migration ability. Therefore, ZNF549 can be used as a new molecular biomarker and cancer therapeutic target to improve the prognosis in COAD patients.

In recent years, more and more evidence demonstrated that abnormal expression and mutation of miRNAs play a key role in tumorigenesis^27^. So far, more than 1,000 miRNAs have been identified, and they are involved in almost all biological processes, including cell proliferation and tumor angiogenesis^28,29^. In colon adenocarcinoma, a number of miRNAs deregulated such as hsa-miR-143, hsa-miR-145, has-miR-21, hsa-miR-200c and let-7a^27,30,31^. Mir-708-5p is an important member of miRNAs and plays an important role in various cancer diseases. Wu et al. reported for the first time that miR-708-5p acts as a direct negative regulator of anti-metastatic miRNA and cyclin-dependent kinase inhibitors in human non-small cell lung cancer^32^. In glioblastoma, the research suggested that miR-708 affects glioblastoma cell proliferation, invasion, and apoptosis through regulating AKt1, CCND1, MMP2, EZH2, Parp-1 and Bcl2^19^. To further investigate the molecular mechanism of ZNF549 regulating COAD progression, we used the TargetScan website (www.targetscan.org) to predict the miRNA targeting of ZNF549. We found that ZNF549 gene transcript might be a potential target gene of miR-708-5p (Fig. 3A). Mir-708-5p negatively regulates the expression level of ZNF549, affecting the proliferation and migration ability of HCT116 and SW620 cells (Fig. 3B–D). In this study, miRNA-708-5p was highly expressed in COAD cells. Experimental results show that miR-708-5p was a promoting factor of COAD cell development. However, in other types of cancer diseases, such as lung cancer, miRNA-708-5p promotes the development of cancer cells. This difference suggests that miR-708-5p may plays different roles in different types of cancer. These results also suggested that miR-708-5p can be used as a potential drug for the treatment of COAD patients.

Epithelial-mesenchymal transition (EMT) is a highly conserved and fundamental process that regulates the formation of tissues and organs and may also be involved in tumor metastasis^33,34^. The loss of E-cadherin and the gain of vimentin are hallmarks of EMT process and have been shown to be correlated with poor prognosis in multiple solid tumor types^35,36^. PI3K/AKt pathway is the main signaling pathway associated with tumor progression and invasion^37^. PI3K/Akt signaling pathway induce EMT, which is an important step for tumor cell migration and invasion^38^. Therefore, our aim is to validate the hypothesis that miR-708-5p promotes COAD progression through the PI3K/AKT pathway. In this study, we also found that miR-708-5p negative regulate ZNF549 to suppress the metastasis of COAD cells by promoting EMT through regulating PI3K/AKT pathway (Fig. 4A–C). However, the detail underlying mechanism for miR-708-5p/ZNF549 molecular axis promoting EMT has not been elucidated. The specific molecular mechanism of miR-708-5p/ZNF549 regulating COAD cell migration needs further study.

Despite significant progress in cancer treatment over the past 50 years, the treatment of metastatic solid cancer remains one of the biggest challenges in modern clinical cancer research. The survival time of patients with this malignant tumor is usually only a few months. Our research represents a new understanding of the molecular mechanism and treatment of COAD.

## Methods and materials

### Clinical data, human COAD cell lines and tissues

Colon adenocarcinoma (COAD) patients and corresponding clinical data, as well as the relative expression levels of *ZNF549* used in this study downloaded from online cancer OMICS database UALCAN (https://ualcan.path.uab.edu/index.html). Human COAD cell lines HT29, HCT116, SW480, LoVo, and SW620, obtained from the Kunming wildlife cell bank of Chinese Academy of Sciences were cultured in 1640 medium (HyClone, Logan, UT, USA) containing 10% fetal calf serum (FCS) (HyClone, Logan, UT, USA) and 1% penicillin/streptomycin and then maintained at 37 °C in an humidified 5% CO_2_ atmosphere. Cells were cultivated in culture flasks at 37 °C in a 5% CO_2_ atmosphere. Cells were used in logarithmic growth phase. Human COAD specimens and adjacent normal tissues were collected from 84 patients who were treated at the Department of General Surgery of Shanghai Eighth People’s Hospital. The research was approved by the Shanghai Eighth People’s Hospital Ethics Committee. The reasonable use of specimens was obtained from written informed consent of patients or their relatives.

### Vector construction and cell transfection

The full-length coding sequence of ZNF549 amplified by PCR using the indicated primers (Supplementary Table S2) and inserted into pcDNA3.1 using Hieff Clone Plus One Step Cloning Kit (Yeasen, Shanghai, China) to construct OE-ZNF549 overexpression plasmid. We synthesized a small interfering RNA (siRNA) that specifically inhibits the expression of ZNF549 (siRNA-ZNF549) and a negative control siRNA (NC) (GenePharma, Shanghai, China). The miR-708-5p mimic, miR-708-5p inhibitor, and miR-NC (control) was constructed and purchased (GenePharma, Shanghai, China) and transfected into cells using Invitrogen Lipofectamine2000 (Life Technologies, NY, USA).

### RNA extraction and quantitative real-time PCR

Total RNA was isolated using TRIzol (Thermo Fisher Scientific, Rockford, USA) and purified with RNeasy mini kit (Qiagen, Hamburg, Germany) according to manufacturer’s instructions. RNA quality and quantity were measured by using nanodrop spectrophotometer and RNA integrity was determined by gel electrophoresis. Human COAD cell lines in the logarithmic growth period were collected. Following the isolation of total RNA, cDNA were synthesized using PrimeScript reagent Kit with gDNA Eraser (Takara, Dalian, China). Quantitative RT-PCR was performed with the cobas z480 real-time PCR instrument (Roche, Basel, Switzerland). Amplification conditions as follows: 96 °C for 20 s, followed by 95 °C for 20 s, and 55 °C 20 s for 40 cycles. Melting curve analysis were used to verify the generation of a single product. *β-actin* was used as a reference gene, and all reactions were repeated three times. Data calculated using the 2^−ΔΔCt^ method^39^. The primers used were shown in Supplementary Table S2.

### CCK-8 assay

The cell proliferation rate were detected using the Cell Counting Kit-8 (Dojindo Molecular Technologies, Kumamoto, Japan) according to the manufacturer’s instructions. The 100 μL of HCT116 and SW620 cells suspension (1000 cells per well) was seeded in 96-well plate. The cells were transfected with normal control (control), miR-708-5p mimics, miR-708-5p inhibitors. The cells were incubated for 24 h and added 10 μL CCK-8 solution. The absorbance was measured at 450 nm using a 96-well microplate reader. All the samples were detected for three times.

### Cell migration and invasion assays

Cell migration and invasion assays were performed using 24-well Transwell chambers.

For the migration assay, The plasmids (OE-ZNF549, siRNA-ZNF549, empty control vector) were transfected into HCT116 and SW620 cells in the logarithmic phase. Then the cells were inoculated into 6-well plates to form a monolayer cells. The monolayer cells were drawn into a straight line with nozzle of a 200 mL pipette. Washed the 6-well plate with PBS, add DMEM medium, and take pictures at 0 h and 24 h. For the invasion assay, the upper chamber was precoated with Matrigel (BD Biosciences, CA, USA), and 100 μL of serum-free medium containing 3 × 10^5^ cells was added. Then 500 μL of DMEM containing 10% FBS was added into the lower chamber. After incubation for 24 h, the cells were fixed with 90% methanol. The number of cells was counted using an IX71 inverted microscope (Olympus, Tokyo, Japan).

### Bioinformatics analysis and luciferase reporter assays

The potential targets miRNAs of ZNF549 was predicted according to TargetScan website (www.targetscan.org). Luciferase reporter plasmids were constructed by Shanghai Jugong Co., Ltd (Shanghai, China). Then HCT116 and SW620 cells were plated into 6-well plates for one night at a fusion density of 70–80% confluence. After incubation, the vector was transfected into HCT116 and SW620 cells using Lipofectamine 2000 reagent (Life Technologies, NY, USA). The method used in the study was previously described by Tobacman^40^. Transfected cells harvested after transfection for 48 h. Dual-luciferase assay system (Promega, WI, USA) used to detect the luciferase activities according to the manufacturer’s protocol. The experiments repeated three times.

### Western blotting

Whole-cell lysates were generated using RIPA lysis buffer (Abcam, Cambridge, UK). Total proteins were separated using 10% SDS-PAGE and then transferred onto a nitrocellulose membrane. The membrane was incubated with the primary antibody at 4 °C overnight, followed by a horseradish peroxidase-conjugated secondary antibody in the second day for 2 h at room temperature. The method used in the study was previously described by Ren^41^. Primary antibodies against the following proteins were used: ZNF549, which was diluted by 1:1000 (Sigma-Aldrich, St. Louis, USA); β-actin, p-PI3K and p-AKt, which were diluted by 1:2000 (Abcam, Cambridge, UK); The corresponding secondary antibodies were diluted by 1:2000 (Abcam, Cambridge, UK). The protein bands were detected with the enhanced chemiluminescence system (ECL) reagent (Pierce, IL, USA). In each experiment, the same amount of protein was used and the experiments were repeated independently at least three times.

### Statistical analysis

GraphPad Prism 8.0 software for windows (www.graphpad.com) (GraphPad Software, San Diego, CA, USA) was used for statistical analysis. Student’s *t*-test were used to analyze the significance between groups by GraphPad Prism 8.0 software. *P*-values of < 0.05 were considered statistically significant. The means ± standard deviation of each group were calculated for all experiments.

### Ethics approval

All procedures performed in studies involving human participants were in accordance with the ethical standards of Shanghai Eighth People’s Hospital Ethics Committee and with the 1964 Helsinki declaration and its later amendments or comparable ethical standards.

### Patient consent for publications

Informed consent was obtained from all individual participants included in the study.

## Supplementary information

Supplementary Information.
