# Supplementary material for: MicroRNA-708 targeting ZNF549 regulates colon adenocarcinoma development through PI3K/AKt pathway
Source: Sci Rep. 2020 Oct 7;10:16729. doi: 10.1038/s41598-020-73929-w (PMC7541523; doi:10.1038/s41598-020-73929-w)
Supplement: Supplementary file 1 — Supplementary Information. [file 41598_2020_73929_MOESM1_ESM.pdf]

MicroRNA-708 targeting ZNF549 regulates colon adenocarcinoma development through PI3K/AKt pathway

Zhidong Zhao[1] Xianju Qin[1]*

1. Shanghai Eighth People's Hospital. Shanghai, China. 200235.

* Authors for correspondence: Xianju Qin

Tel: +86-137 8899 8177

Email: qinxj@hotmail.com

Funding: Major projects of Xuhui district science and technology committee (SHXH201794)

Supplemental Figure S1

Figure 1E

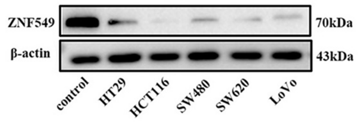

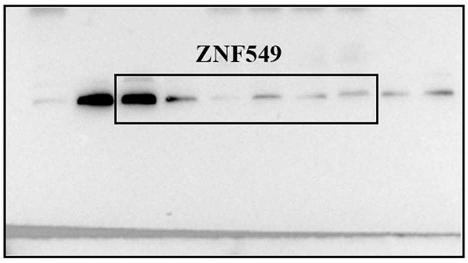

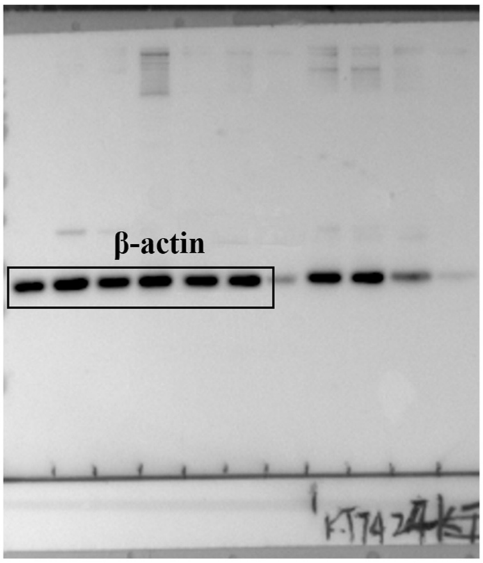

Figure 3C

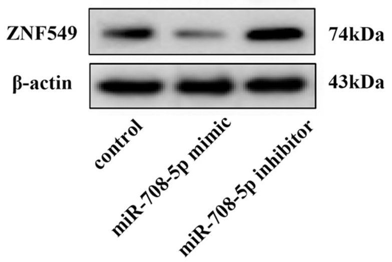
ZNF549
74kDa
β-actin
43kDa
control
miR-708-5p mimic
miR-708-5p inhibitor

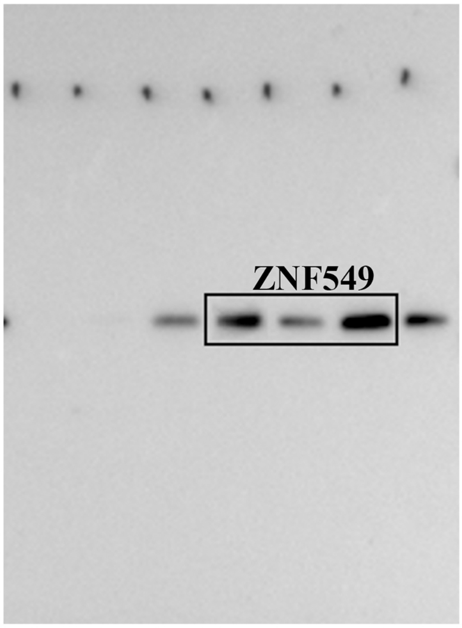
ZNF549

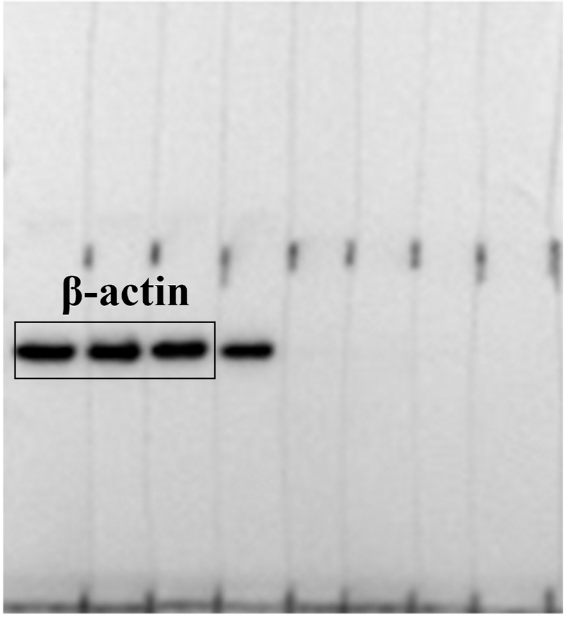
β-actin

Figure 3D

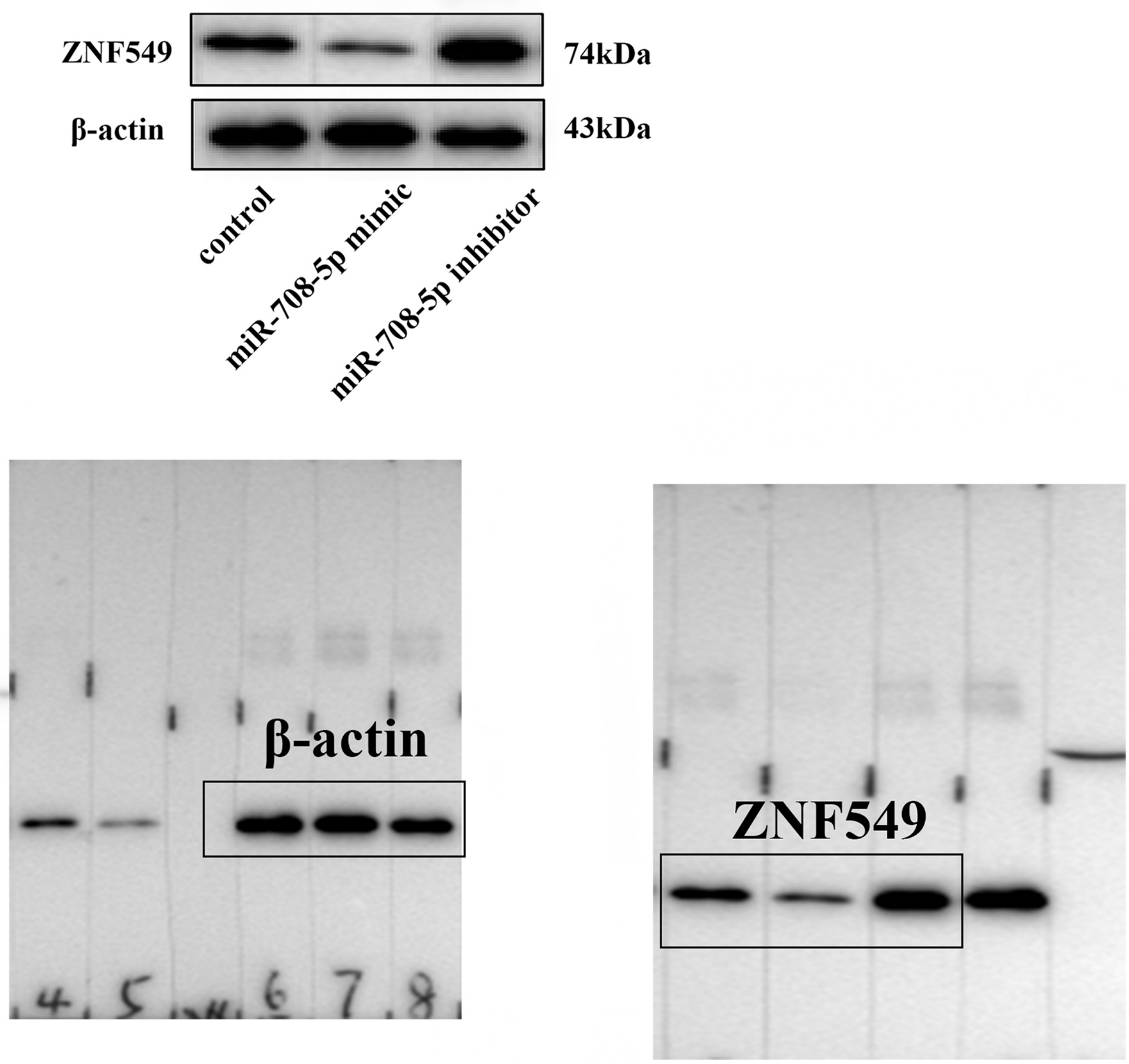
ZNF549
74kDa
β-actin
43kDa
control
miR-708-5p mimic
miR-708-5p inhibitor
β-actin
ZNF549

Figure4B

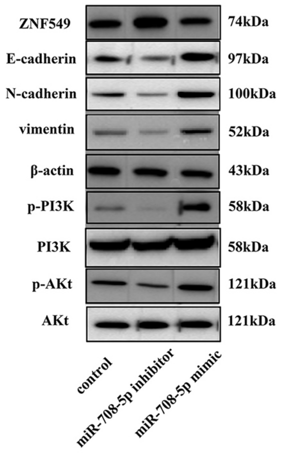

ZNF549
74kDa
E-cadherin
97kDa
N-cadherin
100kDa
vimentin
52kDa
β-actin
43kDa
p-PI3K
58kDa
PI3K
58kDa
p-AKt
121kDa
AKt
121kDa
control
miR-708-5p inhibitor
miR-708-5p mimic

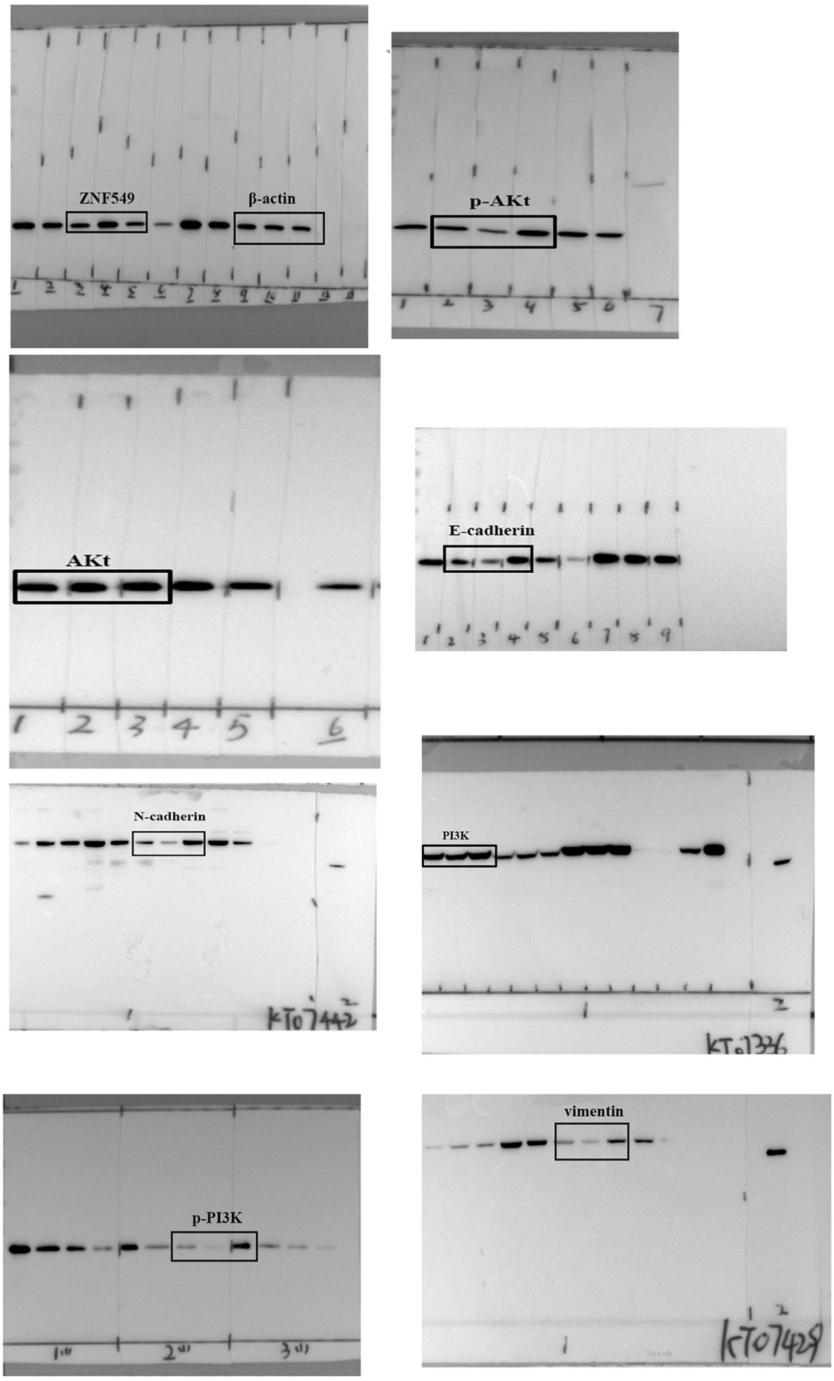

ZNF549
β-actin
p-AKt
AKt
E-cadherin
N-cadherin
PI3K
p-PI3K
vimentin

Supplementary Table S1

Table S1A

Comparision	Statistical significance
Normal-vs-Primary	5.88562532044534E-12

Table S1B

Comparision	Statistical significance
Normal-vs-Stage1	1.6829870802927E-12
Normal-vs-Stage2	2.28509988708936E-10
Normal-vs-Stage3	9.86619999999716E-05
Normal-vs-Stage4	2.458500E-02

Supplementary Table S1

(A) The *P-velue* of normal tissues and tumor tissues. (B) The *P-velue* of normal tissues and tumor tissues based on individual cancer stages.

Supplementary Table 2.

Primers used in this study

Primer name	primer sequence（5' - 3'）
ZNF549-qRCR-F	ATGGCCGAGGCAGCGCTAGTGATTACGCC
ZNF549-qRCR-R	CTAGGGCTCTTCTGTTATATG
miR-708-5p-qPCR-F	GGCGCGCAAGGAGCTTACAATC
miR-708-5p-qPCR-R	GTGCAGGGTCCGAGGTAT
E-cadherin-qPCR-F	TGAAGGTGACAGAGCCTCTGGAT
E-cadherin-qPCR-R	TGGGTGAATTCGGGCTTGTT
N-cadherin-qPCR-F	CACAAGCAGAGTGCTGAAGGTC
N-cadherin-qPCR-R	GATTCCTGAGAGTCCAAAGACAG
Vimentin-qPCR-F	AGATGGCCCTTGACATTGAG
Vimentin-qPCR-R	TGGAAGAGGCAGAAATTC
β-actin-qPCR-F	CTGTGCCCATCTATGAAGGCTA
β-actin -qPCR-R	ATTTCTCTCTCGGCTGTGGTG
